# Data on the influence of ECA implant on microhardness and wear characteristics of composite coating on mild steel

**DOI:** 10.1016/j.dib.2018.11.060

**Published:** 2018-11-15

**Authors:** O.S.I. Fayomi, L. Rudolf. Kanyane, A.P.I. Popoola

**Affiliations:** a Department of Mechanical Engineering, Covenant University, P.M.B. 1023, Ota, Nigeria; b Department of Chemical, Metallurgical and Materials Engineering, Tshwane University of Technology, P.M.B. X680, Pretoria, South Africa

**Keywords:** *Solanum tuberosum* (ST), Microhardness, Wear, Potential difference

## Abstract

In this study, the effect of the incorporation of composite and eco-friendly particles to develop new engineering materials on the developed zinc electrolyte containing TiO_2_/TiB and *Solanum tuberosum* is presented. The electro-depositions were completed at 20 min at a stirring rate of 150 rpm at temperature of 50 °C and pH of 4.The effect of *S. tuberosum* (ST) as bath additive at varied interval of 5–25 ml to the coating properties was noted. Electrodeposition parameters were constant at a voltage of 3.5 V for Zn-based coatings. The outline of bath condition as it influences the microhardness and wear rate were set into consideration. Hence, the coating microhardness and wear rate at constant electrodeposition parameters and varied ST were acquired. Hence, liquid fluid additives can be used for performance of fabricated coatings in advanced surface engineering application.

**Specifications table**

**Table:** 

Subject area	*Materials Engineering*
More specific subject area	*Surface Engineering*
Type of data	*Table, image*
How data was acquired	The electrodeposition was performed using a constructed electrodeposition cell containing two zinc anodes and prepared zinc reach sulphate bath and prepared ST fluid. Mild steel was also prepared via metallography route and co-deposition with zinc based solution at varied ST concentrate. The coating Microhardness and wear rate were measured using Vickers hardness tester machine and MTR-300 abrasive tester respectively.
Data format	Raw, Analysed
Experimental factors	Before electrodeposition, pH was obtained and thermometer was used to confirm the plating temperature. The distance between the anodes and the depth of the samples were measured appropriately before co-depositing the samples.
Experimental features	The electro-depositions of mild steel samples were performed at 20 min at the stirring rate of 150 rpm and temperatures of 50 °C. The effect of coating mechanical properties (Microhardness and wear) were measured at constant voltage of 3.5 V for Zn-TiO_2_/TiB_2_. It was considered that the outline of bath influences in the presence of ST was put into consideration.
Data source location	Department of Chemical, Metallurgical and Materials Engineering, Tshwane University of Technology, Pretoria, South Africa
Data accessibility	Data are available within this article

**Value of the data**

• The given data will give demonstration to surface engineering specialists on the connection and effect of ST on zinc based electrolyte and the continuous metal matrix induced electrolyte in a given engineering components.
• The data could be used for investigating the effect of eco-friendly biodegradable solanum tuberosum juice on the coating microhardness and wear rate progression for advanced engineering application.
• The data obtained can be used in investigating the strengthening behaviour of ST in a zinc based electrolyte relating to its mechanical characteristics.

## 1 Data

The microhardness and wear rate of the fabricated coatings at constant process parameters were collected and the experimental outline of the work data was generated and represented via plots. Electrodeposition took place at 50 °C temperature for 20 min at a stirring speed of 150 rpm. Table 1 shows the chemical composition of the low carbon steel used. The influence of the incorporation of TiO_2_/TiB_2_/ST on wear and microhardness data was collected.

**Table 1:** Chemical Composition of low carbon steel sample used.

Element	C	Mn	Si	P	S	Al	Ni	Fe
Wt %	0.18	0.45	0.18	0.01	0.031	0.005	0.008	99.19

**Fig. 1:**
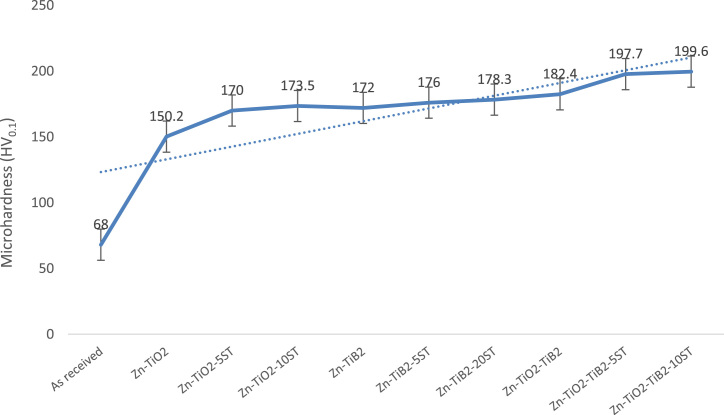
: Experimental trend of variation of TiO_2_/TiB_2_/ST on microhardness properties of mild steel.

**Fig. 2:**
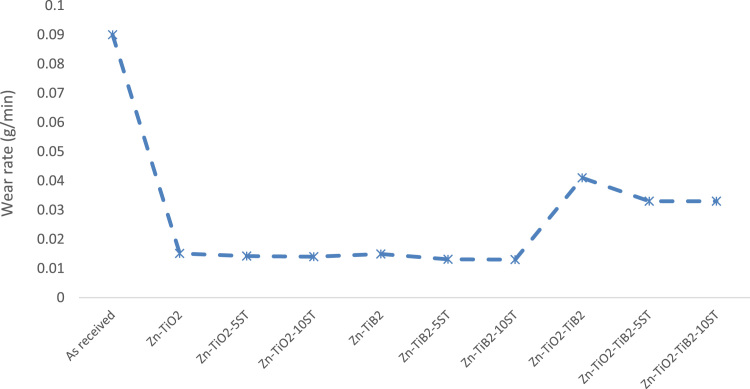
: Experimental trend of variation of TiO_2_/TiB_2_/ST on wear properties of mild steel.

Fig. 1 below shows the microhardness distribution of the electrodeposited Zn–TiO_2_, Zn–TiB_2_ and Zn–TiO_2_–TiB_2_ with variation addition of *Solanum* (5 and 10 L) at constant current density. From the collected data, it was observed that the addition of ST on Zn–TiB_2_ and Zn–TiO2 and Zn–TiO_2_–TiB_2_ composited shows improved microhardness properties. Maximum hardness value of 199.6 HV_0.1_ was obtained at Zn–TiO_2_–TiB_2_–10ST. These highest microhardness properties hence can be linked to the network of natural eco-friendly additives participating at the formation of the coatings [1]. The wear study of Zn–TiO_2_, Zn–TiB_2_ and Zn–TiO_2_–TiB_2_ results were collected with much focus on the addition of *Solanum* additive on the composites coatings. Fig. 2 shows the summarized progression of the mass loss of both coated and control samples. From the collected data, it may be noted that there is considerably reduction in wear plastic deformation of the electrodeposited samples with or without the addition of ST as compared with the control samples. The generated data also shows that the addition of *Solanum tuberosum* gives better enhancement in anti-wear properties which is supported by result observed from [2].

## 2 Experimental design, materials and methods

A plane low carbon steel sheet of 60 mm × 60 mm dimension, with the thickness of 1 mm, was used as a substrate in this research. Other used materials include a zinc plate anode (99.9% pure) and grinding paper in the order of 60 μm, 120 μm, 400 μm, 800 μm, and 1,600 μm for surface preparation. An electrodeposition bath solution was prepared using distilled water. Samples were activated by sinking them into a 2 M HCl solution for 10 s, and then rinsing them in distilled water, which is in accordance with Popoola et al. [3].The electrolyte bath were prepared a day before depositing. Stir took place continuously at the rate of 150 rpm for homogeneity. The bath ad-mixed powders used are represented in Table 2, Table 3. The choice of the deposition parameter is in line with the ones provided in the literature [4], [5], [7]. *S. tuberosum* tuber of equivalent weight of 15 g were selected, peeled, washed, and sectioned into smaller pieces. Then the smaller pieces were squeezed into deionized water to remove the fluid. The mined juice was stored in clean bottles and refrigerated. The *S. tuberosum* tuber used was shopped from Pretoria, South Africa [6].

**Table 2:** Bath composition of Zn–TiO_2_/TiB_2_/*Solanum* and operating condition used.

Composition	Mass concentration (g/L)
ZnSO_4_	80
TiO_2_	20
TiB_2_	20
*Solanum tuberosum*	10–25 (ml)
Boric Acid	15
NaSO_4_	20
Glycine	10
Thiourea	15
Parameters	4
pH	2 V
Voltage	20 min.
Time Temp.	50 °C

**Table 3:** Summarized data of ternary Zn–TiO_2_ /TiB_2_/*Solanum* alloy of electroplated samples.

	**Time of**	**Potential (V)**	**Current**
**Sample order**	**deposition (min)**		**density (A/cm**^**2**^**)**
As received	–		–
Zn–TiO_2_	20	2	3.5
Zn–TiO_2_–5ST	20	2	3.5
Zn–TiO_2_–10ST	20	2	3.5
Zn–TiB_2_	20	2	3.5
Zn–TiB_2_–5ST	20	2	3.5
Zn–TiB_2_–20ST	20	2	3.5
Zn–TiO_2_–TiB_2_	20	2	3.5
Zn–TiO_2_–TiB_2_–5ST	20	2	3.5
Zn–TiO_2_–TiB_2_–10ST	20	2	3.5
